# Hyperthermia promotes M1 polarization of macrophages via exosome-mediated HSPB8 transfer in triple negative breast cancer

**DOI:** 10.1007/s12672-023-00697-0

**Published:** 2023-05-26

**Authors:** Di Xu, Zhen Liu, Ming-Xing Liang, Wen-Quan Chen, Yin‑Jiao Fei, Su-Jin Yang, Yang Wu, Wei Zhang, Jin-Hai Tang

**Affiliations:** 1 Department of General Surgery, The First Affiliated Hospital of Nanjing Medical University, Nanjing, 210029 People’s Republic of China; 2 Department of General Surgery, Taixing People’s Hospital, Taixing, 225400 People’s Republic of China; 3 Department of Biobank, The First Affiliated Hospital of Nanjing Medical University, Nanjing, 210029 People’s Republic of China

**Keywords:** Hyperthermia, M1 macrophages polarization, Exosomes, Triple-negative breast cancer, Heat shock protein family B (small) member 8 (HSPB8)

## Abstract

**Purpose:**

To investigate the mechanism underlying the modulation of M1 macrophage polarization by exosomes released from hyperthermia-treated triple-negative breast cancer (TNBC) cells.

**Materials and methods:**

In this study, the effects of hyperthermia on TNBC cells were examined using cell counting kit-8, apoptosis, and cell cycle assays. Transmission electron microscopy was used to identify the structure of exosomes, while bicinchoninic acid and nanoparticle tracking analysis were used to detect particle size and amounts of exosomes released after hyperthermia. The polarization of macrophages incubated with exosomes derived by hyperthermia-pretreated TNBC cells were assessed by RT-qPCR and flow cytometry analysis. Next, RNA sequencing was performed to determine the targeting molecules changed in hyperthermia-treated TNBC cells in vitro. Finally, the mechanism underlying the modulation of macrophage polarization by exosomes derived from hyperthermia-treated TNBC cells was examined by using RT-qPCR, immunofluorescence and flow cytometry analysis.

**Results:**

Hyperthermia markedly reduced cell viability in TNBC cells and promoted the secretion of TNBC cell-derived exosomes. The hub genes of hyperthermia-treated TNBC cells were significantly correlated with macrophage infiltration. Additionally, hyperthermia-treated TNBC cell-derived exosomes promoted M1 macrophage polarization. Furthermore, the expression levels of heat shock proteins, including HSPA1A, HSPA1B, HSPA6, and HSPB8, were significantly upregulated upon hyperthermia treatment, with HSPB8 exhibiting the highest upregulation. Moreover, hyperthermia can induce M1 macrophage polarization by promoting exosome-mediated HSPB8 transfer.

**Conclusion:**

This study demonstrated a novel mechanism that hyperthermia can induce M1 polarization of macrophages via exosome-mediated HSPB8 transfer. These results will help with future development of an optimized hyperthermia treatment regime for clinical application, especially for combination treatment with immunotherapy.

**Supplementary Information:**

The online version contains supplementary material available at 10.1007/s12672-023-00697-0.

## Introduction

 Globally, breast cancer remains the first leading cause of cancer incidence and the fifth leading cause of cancer mortality in women [1]. Triple-negative breast cancer (TNBC), although accounting for only about 20% of the patient population, TNBC has the poorest outcomes in all subtypes of breast cancer [2, 3]. TNBC is characterized by the lack of estrogen receptor, progesterone receptor, and human epidermal growth factor receptor 2. Owing to tumor heterogeneity and the lack of specific targets, patients with TNBC are prone to metastasis and chemo-resistance [4]. Also, compared with other subtypes, TNBC has unique tumor microenvironment (TME), with higher immune infiltration and immune heterogeneity to help form pre-metastatic niche [5–7]. Therefore, it is urgent to discover novel therapeutic approaches targeting TNBC and its TME.

Hyperthermia therapy, a non-traditional treatment, has been applied as an adjuvant cancer treatment strategy to improve the therapeutic effects of chemotherapy and radiotherapy [8]. A series of clinical trials reported that hyperthermia can enhance complete response rates in recurrent breast cancers after radiation therapy and chemotherapy with minimal acute and late morbidities [9, 10]. Previously, we demonstrated that radiofrequency ablation under the guidance of magnetic resonance can effectively treat residual breast lesions in advanced breast cancer [11]. In contrast to targeted therapy and small molecule inhibitors, hyperthermia therapy is not restricted by the signal transduction pathways of individual cancers, including TNBC. Local hyperthermia treatment can be considered to achieve in situ tumor vaccination via mediating the release of tumor-specific antigens from cancer cells and the production of pro-inflammatory cytokines [12–14]. Hyperthermia has been verified to be able to induce immunogenic cell death of tumor cells and promote CD8 + cytotoxic T lymphocyte infiltration [15]. Thus, an in-depth understanding of the influence of hyperthermia to the immune cells is very necessary.

Tumor-associated macrophages (TAMs) constitute the largest population of immune cells in the TME, which regulates almost all biological processes of the tumor, including tumor progression after hyperthermia treatment [16, 17]. It is mainly divided into M1 type with inflammatory function and anti-tumor activity and M2 type with promoting tumor progression and immunosuppression [16, 18]. It has been found that the increase of TAM and the decrease of M1/M2 TAM ratio in breast cancer have been proved to be associated with poor prognosis [19, 20]. However, the response of TAM to cancer cells in a hyperthermic environment has not been elucidated.

Emerging evidence has revealed that the communication between cells is mediated by exosomes, a type of extracellular vesicles with a diameter of 30–150 nm formed by the cell plasma membrane inwardly budding and the subsequent formation of multivesicular endosomes (MVEs) [21]. Cargos such as RNA and proteins delivered by exosomes in TME can promote immune cascade reaction and induce phenotypic changes of receptor cells [22, 23]. In this study, we investigated the anti-tumor potentials of hyperthermia in TNBC and the effect in exosome secretion. In order to reveal the molecular mechanism involved in the hyperthermia-induced activation of immune responses in TNBC cells, we elucidated the immunogenic role of exosomes derived from hyperthermia-treated TNBC cells and explored their mechanisms in promoting the polarization of TAM.

## Materials and methods

### Cell lines and culture

The human breast cell line MCF-10 A, human breast cancer cell lines (MCF7, T47D, SKBR-3, MDA-MB-231 and Hs578T), human monocyte cell line THP-1 and human umbilical vein endothelial cell line HUVEC were all purchased from the National Collection of Authenticated Cell Cultures (Shanghai, China). T47D, SKBR3, MCF7, MDA-MB-231, Hs578T and HUVEC cells were cultured with DMEM medium (Procell, China) containing 10% fetal bovine serum (FBS). THP-1 cells were cultured with RPMI-1640 medium (Procell, China) with 10% FBS and 0.05 mM ß-Mercaptoethanol. MCF-10 A cells cultured with MEGM kit (Lonza Clonetics, Switzerland). All cells were cultured at 37 °C and in 5% CO_2_ humidified atmosphere incubator.

### Hyperthermia treatment

The hyperthermia treatment conditions were as follows: temperature, 43 °C; duration, 1 h. Briefly, cells were allowed to adhere in a culture flask in advance. After reaching the logarithmic phase, the cells were incubated at 43 °C for 1 h in incubator. To reduce the time of temperature rise, we prepared a 43 °C pre-heated medium and replace the original medium. Control group cells were maintained in a 37 °C medium. After hyperthermia treatment, the cells were transferred to a 37 °C incubator. Further examinations were conducted after 6 h of recovery.

### Cell counting Kit-8 (CCK-8) assay

Cells were seeded at an optimized concentration of 5000 cells/well in sterile 96-well flat-bottom culture plates. Five secondary holes were then arranged in each group. Cells were exposed to hyperthermia treatment (43 °C for 1 h), followed by a recovery period to 6 h. These groups were measured with CCK-8 reagent (APExBio, Houston, USA) and the absorption value at 450 nm was measured on 0, 24, 48, 72 h using Multiskan FC microplate Reader (Thermo Fisher Scientific, USA).

### Cell apoptosis assay and cell cycle assay

After 24 h of hyperthermia treatment, cells were collected. For apoptosis assay, cells were resuspended in 300 µl of 1×binding buffer with 3 µl of Annexin V-FITC and 3 µl of PI for 15 min in the darkness. For cell cycle assay, cells were fixed with 75% ethanol at 4 °C overnight. Then, cells were collected and hydrated with PBS for 15 min, followed by resuspended in 300 µl of PI staining binding buffer (Vazyme, Nanjing, China) for 30 min in the darkness. The percentage of apoptotic cells and cell cycle was detected by using Cytoflex flow cytometer (Beckman, USA).

### Exosome isolation

Exosomes were isolated using gradient centrifugation, following general protocols. After hyperthermia treatment, cells were cultured with DMEM medium containing 10% exosome-depleted FBS for 24 h. Next, the culture medium was collected and centrifuged at 300 g for 10 min, followed by centrifugation at 2000 g for 15 min and 12,000 g for 30 min in turn. The supernatant was then filtered through a 0.22 µm filter and ultracentrifuged at 100,000 g (Beckman Type 90 Ti) for 2 h twice. The exosomes samples were resuspended in PBS for analysis.

### Transmission electron microscopy (TEM)

The isolated exosomes were allowed to adsorb onto a copper network for 2 min. The sample was washed with distilled water and negatively stained with uranium acetate for 2 min. The precipitate of cells was fixed with 2.5% glutaraldehyde at 4 °C overnight. All samples were gradually dehydrated in ethanol. The samples were embedded and sectioned. The section was subjected to double staining with 3% uranyl acetate-lead citrate. Transmission electron microscope (JEM-1010, JEOL, Japan) was used to observe the structure of exosomes.

### Total protein extraction and Western blot (WB)

Total protein of cells and isolated exosome was extracted using phenylmethanesulfonyl fluoride (PMSF) diluted 1:100 in cell lysis buffer (Beyotime, Shanghai, China) for 30 min on ice. The protein concentration was measured using a bicinchoninic acid (BCA) protein assay kit (Beyotime, Shanghai, China). After centrifuging at 12,000 ×*g* for 20 min, the supernatant was mixed with 5 × loading buffer (NCM Biotech, Suzhou, China) and then denatured at 99 °C for 10 min.

Equivalent concentration proteins of cells and exosomes was carefully loaded in each lane of a 10% SDS-polyacrylamide gels by electrophoresis and transferred onto a 0.45 µm PVDF membrane (Millipore, USA). Then, the membranes were blocked with QuickBlock^™^ Blocking Buffer for Western Blot (Beyotime, Shanghai, China) for 20 min. The membranes were incubated overnight at 4 °C with primary antibodies CD63 antibody (1:1000, Abcam, ab68418), TSG101 antibody (1:1000, Abcam, ab125011) and Calnexin antibody (1:1000, Abcam, ab133615). after washed, the membranes were incubated with species-specific secondary antibody at room temperature for 1 h. Finally, the proteins were detected by the BeyoECL Plus kit (Beyotime, Shanghai, China) under ChemiDoc^™^ Touch Imaging System (Bio-Rad, USA).

### Nanoparticle tracking analysis (NTA)

ZetaView^®^ PMX120 nanoparticle tracking analyzer was used to analyze the size distribution and concentration of exosomes. Exosome samples were diluted in PBS and pumped for analysis.

### Cell differentiation induction

To promote the differentiation of monocytes into M0 macrophages, THP-1 cells were cultured in complete medium with 100 ng/mL PMA (Sigma-Aldrich, USA) for 48 h to promote the differentiation of monocytes into M0 macrophages. To promote the activation of M0 macrophages into M1 macrophages, the cells were incubated with preliminary medium containing 100 ng/mL LPS (Sigma-Aldrich, USA) instead of 1640 complete medium for 48 h.

### Cellular uptake of exosomes

The uptake of PKH26-labeled exosomes by recipient cells was examined using confocal microscopy. The 5000 recipient cells were seeded in a confocal dish. Exosomes were labeled using the PKH26 red fluorescent labeling kit (Sigma-Aldrich, USA) following the manufacturer’s instructions. The recipient cells were co-cultured with 20 µg exosomes for 24 h. Next, the cells were washed and fixed with 4% polyformaldehyde for 30 min, followed by staining with Actin-Tracker Green (1:1000, Beyotime, China) and DAPI (1:1000, Beyotime, China) for 30 min in the dark. The uptake of the dye was examined using a Stellaris STED laser confocal microscope (LEICA, Germany).

###  Total RNA extraction and real-time polymerase chain reaction (RT-qPCR)

Total RNA extracted from exosomes using a Trizol ls Reagent (Thermo Fisher, USA). Total RNA extracted from breast cancer cells and macrophages using a total RNA Kit (Tian Gen, DP419). The concentration and purity of RNA were detected by a NanoDrop2000 ultraviolet spectrophotometer (Thermo Fisher, USA). The procedure of RT-qPCR is the same as prviously described [24]. All primers were shown in Supplementary Table 1.

### Flow cytometry analysis of macrophages

To detect the protein markers of macrophages, cells were labeled with CD14-FITC, CD86-APC and CD163-PC7 human antibodies (Biolegend, USA). Briefly, pretreated macrophages were digested using PBS containing 2.5 mM EDTA. The digestion was stopped using 0.5% BSA in PBS. The precipitate was resuspended and incubated in 100 µL PBS with 5 µL human FcR blocking agent (Biolegend, USA) for 15 min to block Fc receptor. After washing, the cells were resuspended in 300 µL of 1× binding buffer with 3 µL CD14-FITC, 3 µL CD86-APC, and 3 µL CD163-PC7 antibodies for 30 min to stain cell surface markers. The percentage of M1/M2 macrophage cells was quantified using a Cytoflex flow cytometer (Beckman, USA).

### RNA-Seq

After hyperthermia exposure, total RNA was extracted and quantified. MDA-MB-231 cells were subjected to genome-wide gene expression analysis using RNA deep sequencing. Library construction was performed by Illumina. The raw data were analyzed with R language. Resulting sequencing files were publicly available at PRJNA957775 of SRA database. Gene Ontology (GO), Kyoto Encyclopedia of Genes and Genomes (KEGG) pathway analysis, and Reactome pathway analysis of differentially expressed genes (DEGs) were performed to identify the function enrichment using DAVID analysis (http://david.ncifcrf.gov).

### Bioinformatics analysis

The protein-protein interaction (PPI) network was constructed using STRING (http://string-db.org) analysis. The hub genes in the PPI network were calculated by method MCC. The expression levels of hub genes in breast cancer datasets of The Cancer Genome Atlas (TCGA) database was analyzed using GEPIA online software (http://gepia.cancer-pku.cn). The correlation between the expression level of hub genes and overall survival (OS) in the datasets of breast cancer retrieved from TCGA database was analyzed using the Kaplan–Meier plotter online software (http://kmplot.com/analysis/). The correlation between the expression levels of hub genes and the degree of macrophage infiltration in breast cancer was analyzed using TIMER2.0 online software (http://timer.cistrome.org).

### Cell transfection

Macrophage cells were transfected with negative control (NC) or predesigned overexpression (OE) plasmid pcDNA3.1-HSPB8 using Hieff Trans® liposomal transfection reagent (Yeasen, Shanghai, China). Meanwhile, MDA-MB-231 and Hs578T cells were transfected with NC or predesigned si-HSPB8 using Lipo8000 transfection reagent (Beyotime, Shanghai, China). Briefly, cells were planked with the density at transfection was 70% in advance. Then, prepare two tubes of mixed reagent. A: 250 µL Opti-MEM + 2.5 µg DNA/100 pmol siRNA; B: 250 µL Opti-MEM + 5 µL transfection reagent. After 5 min, we mixed two tubes and co-cultured for 20 min. Finally, the compound was added to each well for 48 h before further examination.

### Immunofluorescence (IF)

The transfected macrophages were fixed with 4% polyformaldehyde for 25 min. Next, the cells were blocked with QuickBlock^™^ Blocking Buffer (Beyotime, Shanghai, China) for 20 min, and then staining with anti-CD86 antibodies (1:500, Abcam, ab239075) overnight at 4 °C. Then, Cells were stained with species-specific goat anti-rabbit IgG H&L (Alexa Fluor 488) (1:2000, Abcam, ab150077) secondary antibodies for 1 h at room temperature, followed by stained with 30 µL anti-fluorescence quenching sealing agent (including DAPI) (Soleibao, Beijing, China) and covered with the cover glass. After completely drying, Thunder Imager fast high-resolution inverted fluorescence imaging system (LEICA, Germany) was used to observe the expression of CD86.

### Statistical analysis

All experiments were independently performed at least in triplicates, error bars represented the mean ± SD of experiments. All assays were analyzed using t-test of means between two groups of independent samples, and using one-way ANOVAs between multiple groups. Differences were considered significant at p < 0.05, with *p < 0.05, **p < 0.01, ***p < 0.001, ****p < 0.0001.

## Results

### Hyperthermia decreases the viability of TNBC cells and regulates cell apoptosis and cell cycle

**Fig. 1:**
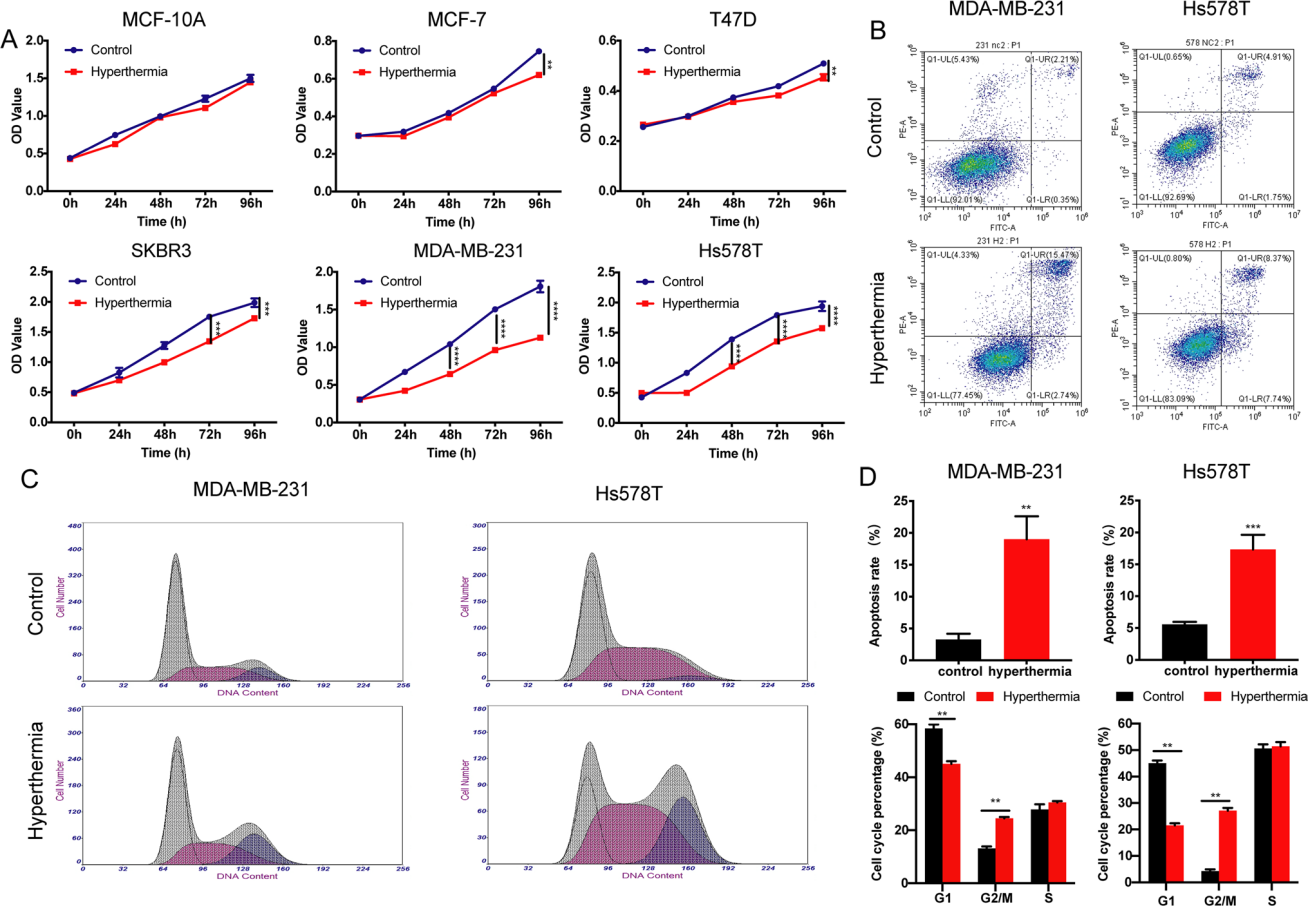
Effect of hyperthermia on breast cancer cells. **A** Normal breast cells (MCF-10 A), Luminal type human breast cancer cells (MCF-7, T47D), Her2 positive human breast cancer cells (SKBR-3) and TNBC cells (MDA-MB-231, Hs578T) were treated with 43 °C water bath for 1 h. Cell viability was measured by using the CCK-8 assay. **B** Cell apoptosis analysis of MDA-MB-231 and Hs578T cells treated with or without hyperthermia were performed using flow cytometry assay. **C** Cell cycle of MDA-MB-231 and Hs578T cells were analyzed using flow cytometry assay before and after hyperthermia treatment. **D** The statistical data of cell apoptosis and cell cycle using GraphPad Prism 8.0

To investigate the biological effects of hyperthermia, the viability of different types of breast cancer cell lines was analyzed by the CCK-8 assay. As shown in Fig. 1A and Supplement Fig. 1, hyperthermia greatly inhibits the proliferation of TNBC cells rather than in other cell types, while showing no cytotoxic effect on normal breast cells MCF-10 A and several different non-tumor cell lines such as HUVEC and macrophages. Then, the effect of hyperthermia on TNBC cell apoptosis and cell cycle stage was examined by using flow cytometry. The percentage of apoptotic TNBC cells was significantly increased after hyperthermia treatment both in MDA-MB-231 and Hs578T cells (Fig. 1B, D). Also, hyperthermia treatment accumulated cells in the G2/M phase, with a concomitant decrease of the G1 phase of the cell cycle (Fig. 1C–D), suggesting that hyperthermia arrests the cell cycle of TNBC cells at G2/M phase.

### Hyperthermia promotes the secretion of exosomes released by TNBC cells

**Fig. 2:**
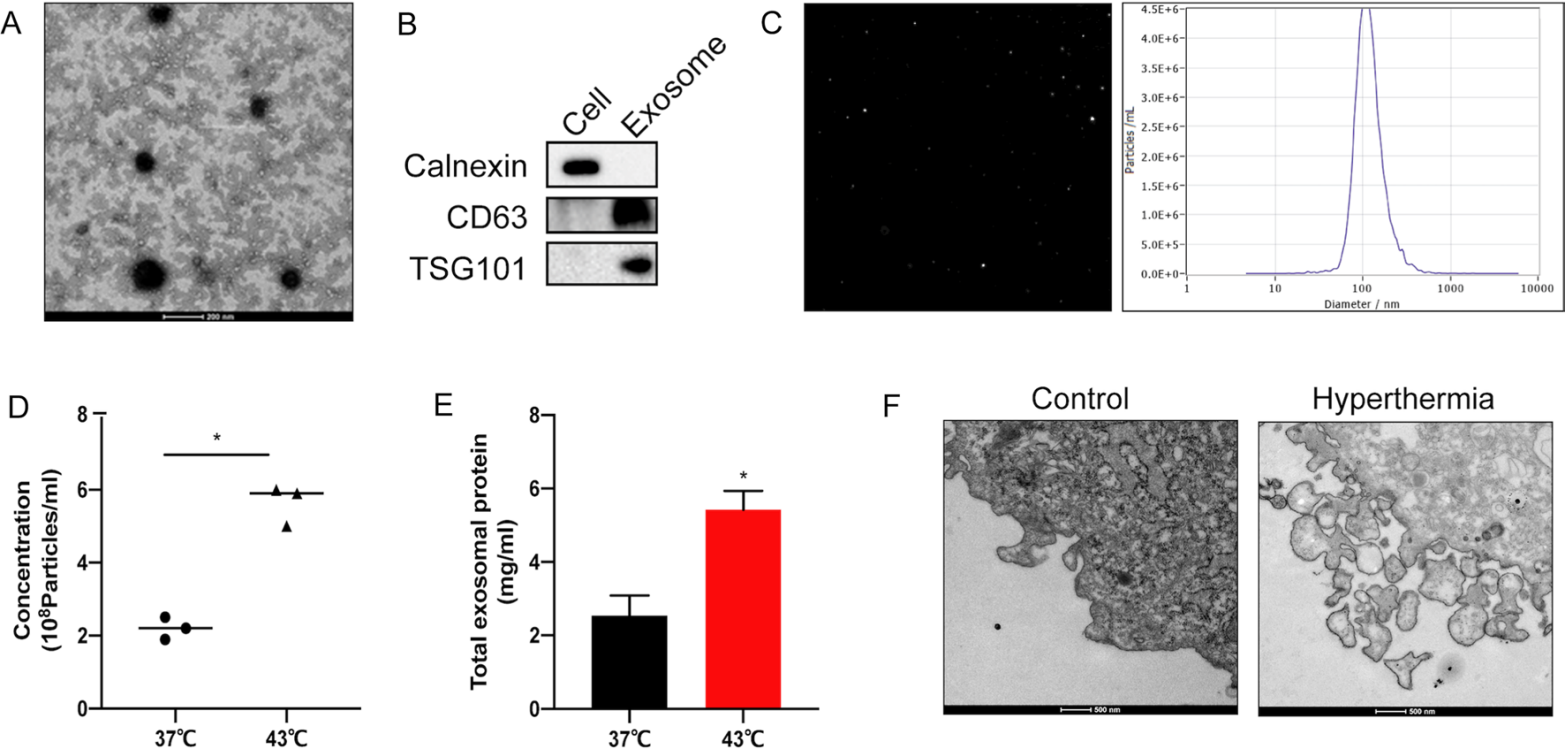
Hyperthermia promotes exosome secretion in MDA-MB-231 cells. **A** Identification the structure of exosomes isolated from MDA-MB-231 cells medium by TEM, Scale bar = 200 nm. **B** Identification of exosomal marker proteins by Western blotting. **C** Size analysis of exosomes by NTA. **D** Pre-treated MDA-MB-231 cells cultured in DMEM medium containing 10% exosome-depleted FBS for 24 h. Particles concentration analysis of exosomes by NTA. **E** The amount of total proteins in exosomes secreted from pre-treated MDA-MB-231 cells were tested by a BCA protein kit. **F** Secretory structure of vesicles of pre-treated MDA-MB-231 cells by TEM, Scale bar = 500 nm

To identify the effect of hyperthermia on the exosomes secretion derived from TNBC cells, hyperthermia-treated MDA-MB-231 cells were subcultured in medium containing 10% exosome-depleted FBS for 24 h. Isolated exosomes using gradient centrifugation were subjected to TEM, western blotting, and NTA to identify the structure, marker proteins, and particle size of exosomes, respectively. As shown in Fig. 2A, TEM analysis revealed a scattered lipid bilayer structure with a size of approximately 100 nm. Western blotting revealed that the membrane structural proteins CD63 and TSG101 were downregulated in the cells but upregulated in the exosomes. In contrast, the expression of the endoplasmic reticulum protein Calnexin was upregulated in cells but downregulated in exosomes (Fig. 2B). NTA revealed that the average particle size of the isolated exosomes was approximately 115.2 nm (Fig. 2C).

Then, the particle size, quantity and protein amounts of exosomes released after hyperthermia were compared by NTA and a BCA protein kit. The concentration levels of exosomes were normalized to cell numbers. The secretion of exosomes from MDA-MB-231 cells was upregulated after hyperthermia, with a slight but no significant increase in particle size (Fig. 2D–E). TEM images (Fig. 2F) revealed enhanced secretory structure of vesicles and intense extracellular vesicle shedding at 6 h post-hyperthermia treatment.

### Exosomes secreted by hyperthermia-treated TNBC cells can regulate macrophage polarization

**Fig. 3:**
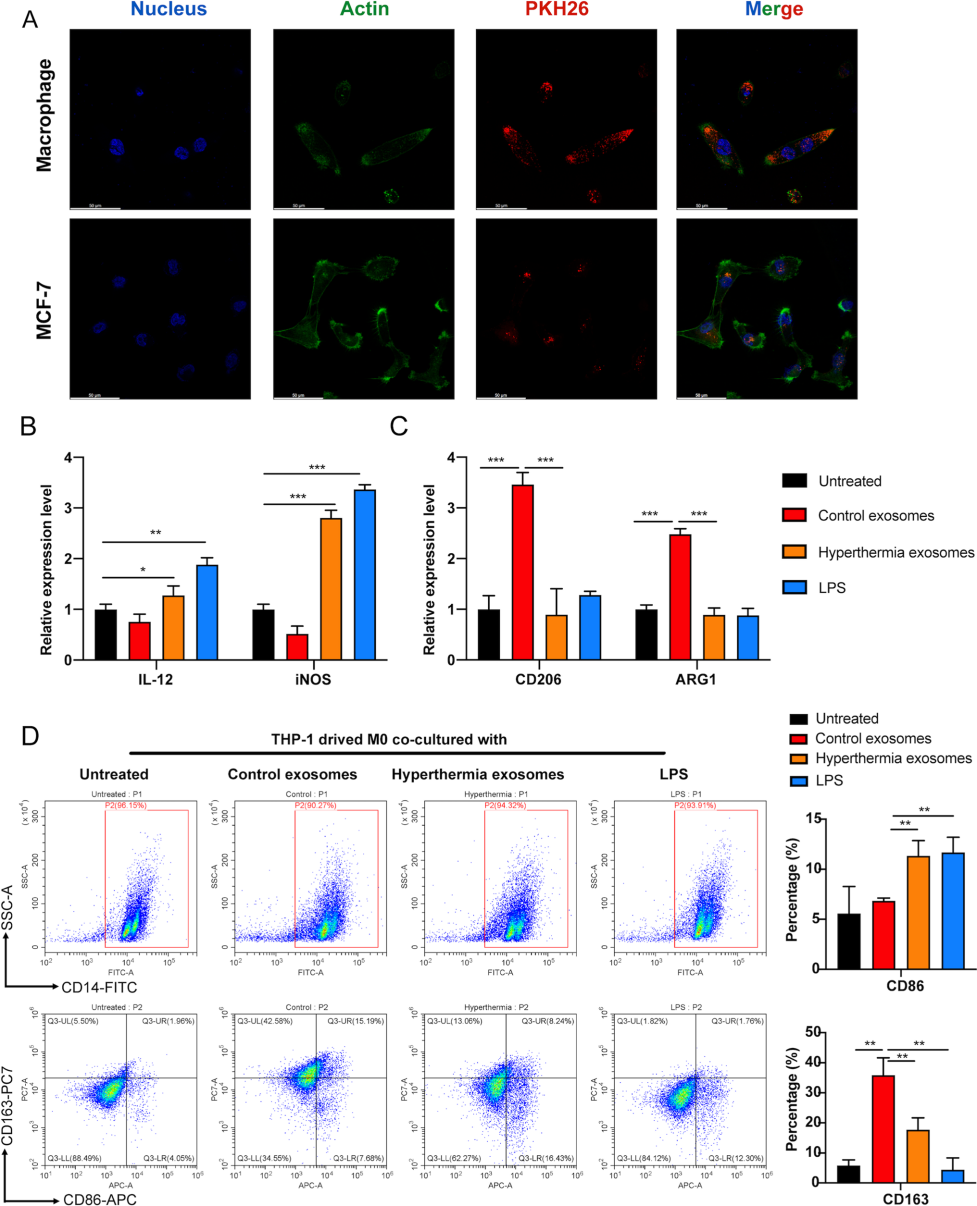
Exosomes secreted by hyperthermia TNBC cells can promote M1 macrophage differentiation. **A** Uptake analysis of exosome by macrophage cells and breast cancer MCF-7 cells by confocal microscope, Scale bar = 50 μm. **B** Taking LPS (100 ng/ml)-induced M1 type macrophages as positive control, the expression levels of M1 markers (IL-12 and iNOS) in macrophages co-cultured with exosomes were tested by RT-qPCR. **C** The expression levels of M2 markers (CD206 and ARG1) in macrophages co-cultured with exosomes were tested by RT-qPCR. **D** The proportions of CD14 + CD86 + M1 macrophages and CD14 + CD163 + M2 macrophages were analyzed by flow cytometry

THP-1 monocytes were used to explore the effect of hyperthermia TNBC cell-derived exosomes on macrophage polarization. First, we investigated the cellular uptake of exosomes in THP-1-induced M0 macrophage cells. As shown in Fig. 3A, we observed enhanced intracellular red fluorescence signals in M0 macrophages after co-incubating with PKH26 labeled exosomes secreted by hyperthermia-treated MDA-MB-231 cells. To exclude the effect that the uptake of exosomes derived from recipient cells themselves was energy-dependent, rather than depending on the recipient cell type [25], we then chose MCF-7 cells to evaluate the uptake ability. The uptake ability of exosomes by macrophages was higher than that of cancer cells in the TME. Next, the polarization-related changes at the transcript level were examined using RT-qPCR analysis of the M1 and M2 phenotypic markers. LPS (100 ng/mL)-induced M1 type macrophages served as the positive control. The mRNA levels of M1 markers (IL12 and iNOS) both upregulated in macrophages co-cultured with exosomes derived from hyperthermia-treated MDA-MB-231 cells compared with those in control exosomes (Fig. 3B). Additionally, the mRNA levels of the M2 markers (CD206 and ARG1) were significantly downregulated in the hyperthermia-treated group but upregulated in the LPS-treated group (Fig. 3C). Furthermore, the proportion of CD86 (M1 marker) and CD163 (M2 marker) on the surface of macrophages in the co-culture group was examined using flow cytometry. The proportion of M1 macrophages (CD14 + CD86+) was upregulated when THP-1 cells were co-cultured with hyperthermia group exosomes, while that of M2 macrophages (CD14 + CD163+) was downregulated (Fig. 3D). This indicates that exosomes released after hyperthermia treatment can promote the M1 polarization of macrophages. Next, the underlying molecular mechanisms will be examined from the aspect of exosome-mediated transcription factor transfer.

### Genome-wide gene identification of TNBC cells treated with hyperthermia

**Fig. 4:**
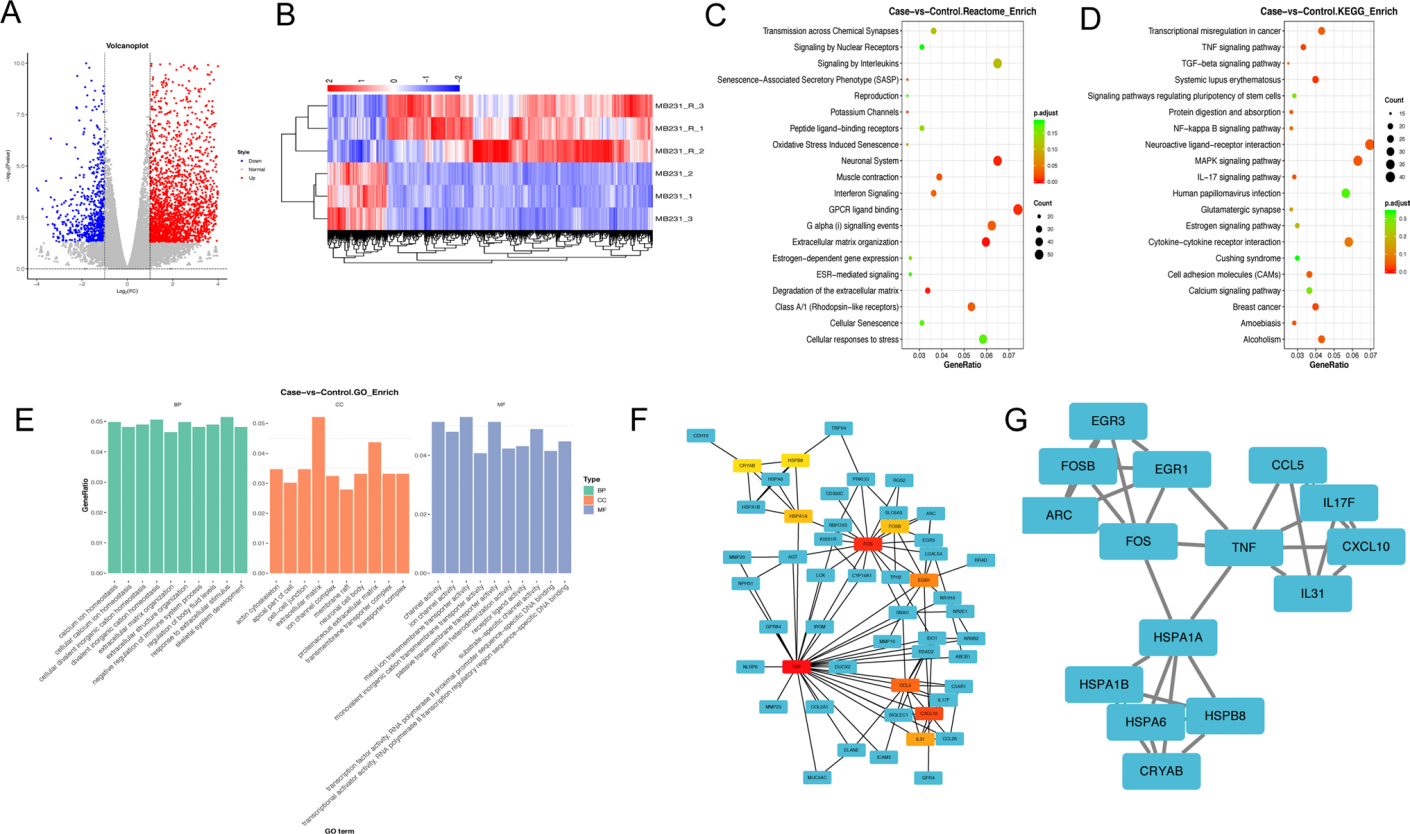
Analysis of RNA-seq data of TNBC cells treated with hyperthermia. **A**–**B** Scatterplot and heat map of DEGs between control and hyperthermia groups. Red color represents up-regulated genes, blue represents down-regulated genes. **C** The Reactome pathway enrichment analysis of DEGs (|log2FC|>1 and P value < 0.05). **D** The KEGG pathway enrichment analysis of DEGs (|log2FC|>1 and P value < 0.05). **E** The GO enrichment analysis of molecular function, biological process and cellular component in DEGs (|log2FC|>1 and P value < 0.05). **F** The PPI network was constructed by DEGs (FC > 2 and P value < 0.05). **G** The most significant module of PPI network

To explore the mechanisms of hyperthermia in TNBC, RNA-seq analysis was performed to identify the genome-wide gene profiles in hyperthermia-treated MDA-MB-231 cells. Volcano map and heat map revealed 3091 upregulated genes and 688 downregulated genes in the hyperthermia-treated cells (Fig. 4A–B). The top 3 identified upregulated DEGs were HSPA6, CRYAB and TDRG1 (Supplement:  Tables 2, 3). The Reactome database was used to analyze DEG-related metabolic pathways and signal transduction pathways. The Reactome database analysis and GO analysis revealed the significantly enriched biological process of DEGs, including extracellular structure organization and negative regulation of cytokine production (Fig. 4C, E). Meanwhile, the DEGs were significantly enriched in the MAPK, TNF, and IL-17 signaling pathways by KEGG pathway analysis (Fig. 4D). Furthermore, we constructed a PPI network to explore hub genes changed in the procedure of hyperthermia (Fig. 4F–G). The top 15 genes (degrees ≥ 5) are shown in Table 1.

**Table 1:** List of the top 15 hub genes after hyperthermia treatment in the PPI network, which were calculated by method MCC.

No.	Gene	Description	Style	Degree	log2FC	P value
1	TNF	Tumor necrosis factor	Up	96	6.327454491	1.36E-13
2	FOS	Fos proto-oncogene, AP − 1 transcription factor subunit	Up	53	6.464266453	2.55E-75
3	CXCL10	C-X-C motif chemokine ligand 10	Up	49	5.330646742	1.01E-06
4	CCL5	C-C motif chemokine ligand 5	Up	44	6.062745054	2.78E-07
5	EGR1	Early growth response 1	Up	37	7.263225519	4.24E-22
6	IL31	Interleukin 31	Up	30	6.100491976	3.35E-05
7	FOSB	FosB proto-oncogene, AP − 1 transcription factor subunit	Up	26	8.553101371	5.63E-56
8	HSPA1A	Heat shock protein family A (Hsp70) member 1 A	Up	26	8.00080639	1.00E-54
9	HSPB8	Heat shock protein family B (small) member 8	Up	25	10.55905491	1.34E-11
10	CRYAB	Crystallin alpha B	Up	25	10.5030895	1.79E-146
11	HSPA6	Heat shock protein family A (Hsp70) member 6	Up	24	12.34220903	4.54E-45
12	ARC	Activity regulated cytoskeleton associated protein	Up	24	8.836556832	5.51E-157
13	IL17F	Interleukin 17 F	Up	24	5.75885765	0.00048
14	HSPA1B	Heat shock protein family A (Hsp70) member 1B	Up	23	6.946689163	3.84E-45
15	EGR3	Early growth response 3	Up	23	6.423293135	1.78E-10

### Hyperthermia-induced hub genes in TNBC cells were correlated with macrophage polarization

**Fig. 5:**
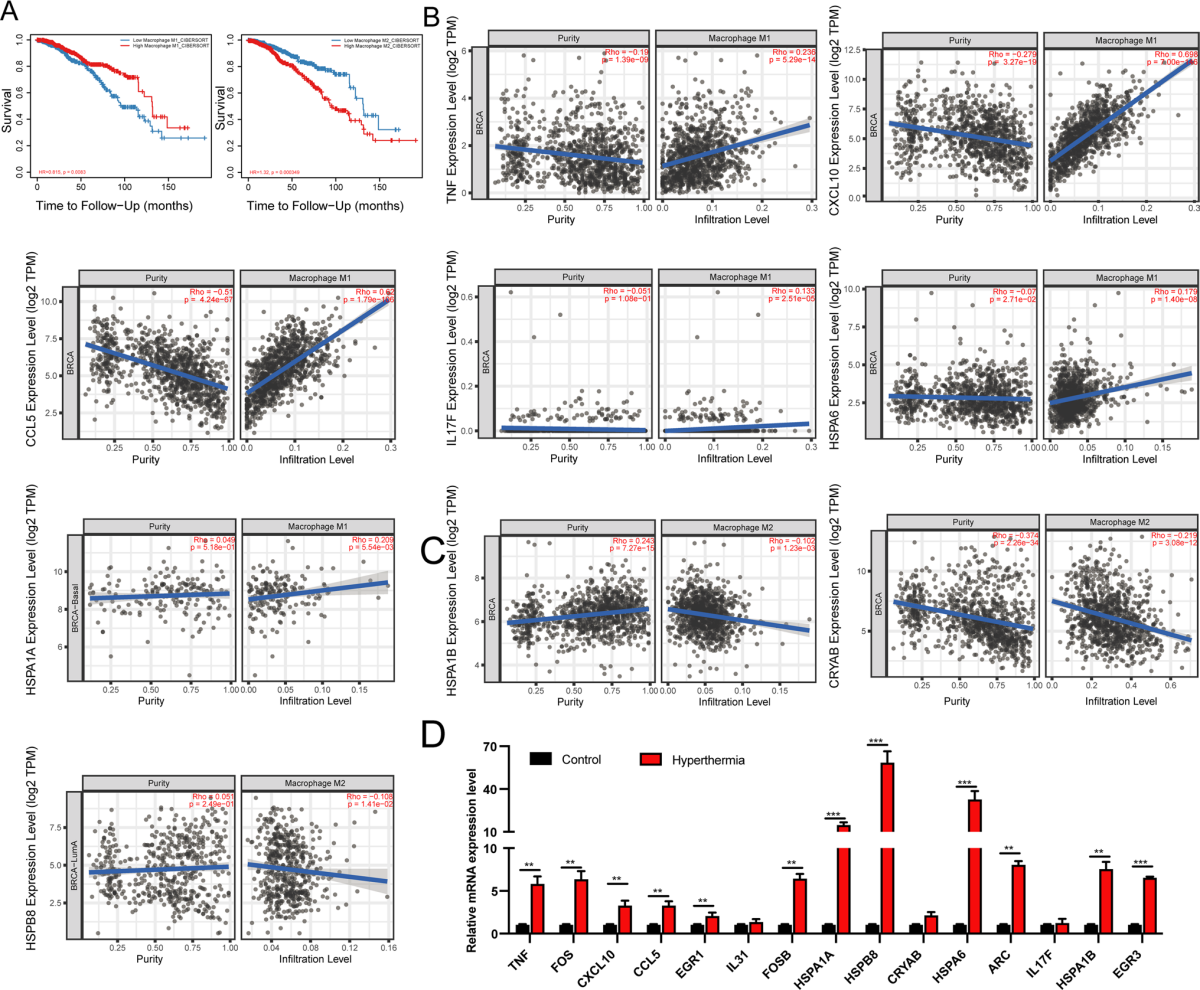
The validation of correlation between hub genes and macrophages. **A** Overall survival analysis of macrophages infiltrate in breast cancer. **B**, **C** The correlation of hub genes expression with macrophages infiltration by TIMER2.0 database. **D** The expression levels of hub genes were verified by RT-qPCR.

In the breast cancer microenvironment, we found that the more M2 macrophages infiltrate, the worse the OS rate of breast cancer patients (Fig. 5A). Therefore, reprogramming M2 macrophages into M1 type is an important method for targeted macrophage therapy. To identify the mechanism of exosomes released by hyperthermia-treated cells in regulating macrophage reprogramming, the correlation between hub genes and macrophages was examined. As shown in Fig. 5B, TNF, CXCL10, CCL5, IL17F, HSPA1A and HSPA6 expression levels were positively correlated with M1 macrophage infiltration. Meanwhile, the expression levels of CRYAB, HSPA1B, and HSPB8 were not correlated with the degree of M1 macrophage infiltration but were significantly and negatively correlated with M2 macrophage infiltration (Fig. 5C). The transcript levels of 15 hub genes in independently collected samples were evaluated using RT-qPCR (Fig. 5D). The expression levels of heat shock protein (HSP) family members, including HSPA1A, HSPA1B, HSPA6, and HSPB8, made significantly increasing after hyperthermia, with HSPB8 exhibiting the highest upregulation.

Additionally, the expression levels of FOS, FOSB, EGR1, and EGR3 were significantly increased and positively correlated with M2 macrophages infiltration (Supplemental Fig. 2A). However, patients with breast cancer exhibiting upregulated expression levels of FOS, FOSB, EGR1, and EGR3 were associated with improved prognosis. The FOS, FOSB, EGR1, and EGR3 expression levels in healthy breast tissues were higher than in breast cancer tissues (Supplemental Fig. 2B–C). Therefore, we hypothesized that FOS, FOSB, EGR1, and EGR3 mainly function as tumor suppressor genes to inhibit the occurrence and progression of breast cancers.

### Exosome-mediated HSPB8 released by TNBC cells under hyperthermia modulates polarization in macrophages

**Fig. 6:**
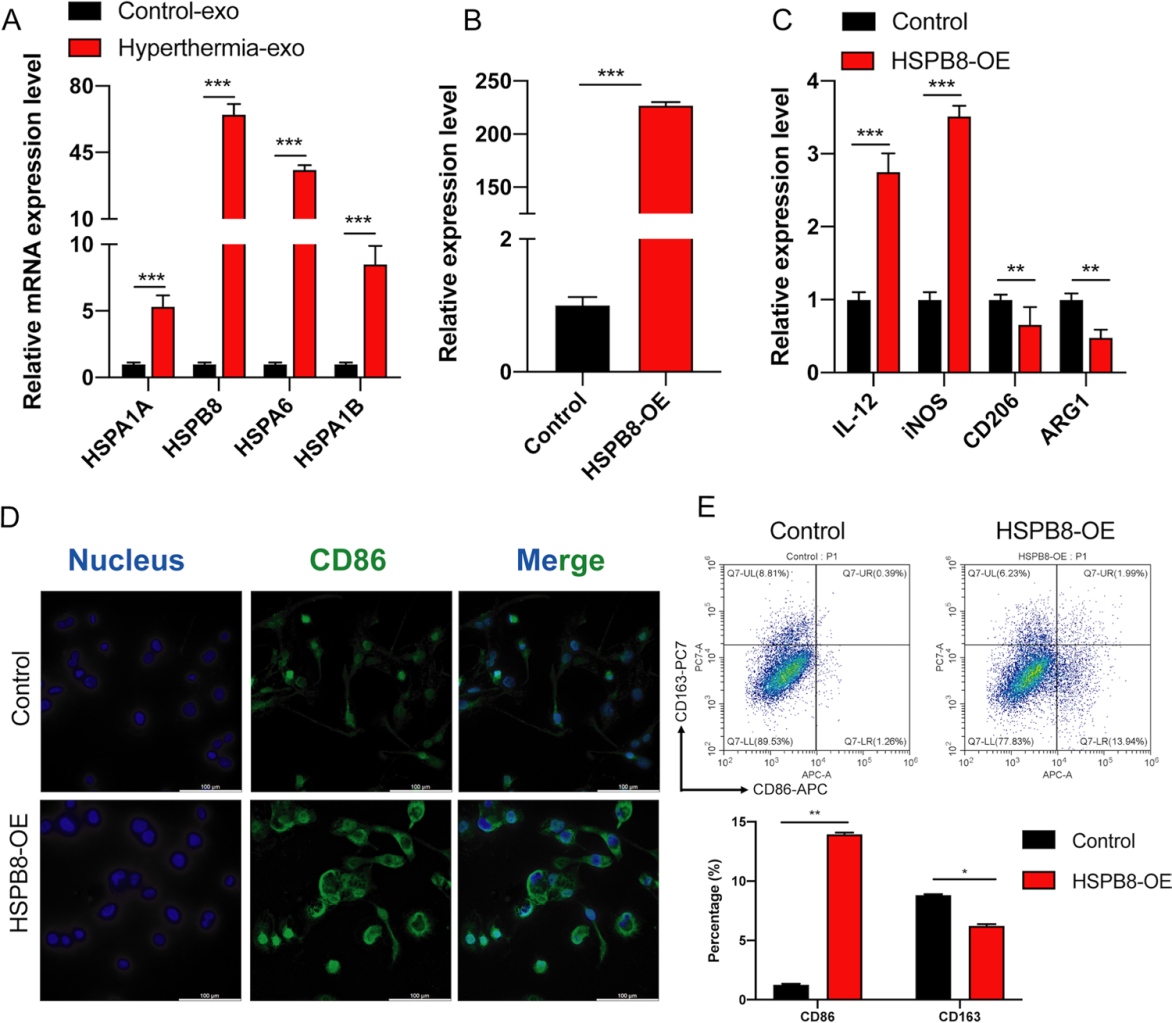
Exosome-mediated HSPB8 released by TNBC cells under hyperthermia modulates polarization in macrophages. **A** The expression of HSPA1A, HSPA1B, HSPA6 and HSPB8 in exosomes released by MDA-MB-231 after hyperthermia. **B** The transfection efficiency of HSPB8 OE plasmids were tested by RT-qPCR. **C** The expression levels of M1 markers (IL-12 and iNOS) and M2 markers (CD206 and ARG1) in macrophages transfected with HSPB8-OE were tested by RT-qPCR. **D** Detection of CD86 expression in macrophages transfected with HSPB8-OE by immunofluorescence assay, Scale bar = 100 μm. **E** The proportions of CD86 + M1 TAM and CD163 + M2 TAM were analyzed by flow cytometry

To clarify the mechanisms underlying the modulation of M1 macrophage polarization by exosomes released from hyperthermia-treated TNBC cells, we first detected the mRNA expression in exosomes. RT-qPCR analysis showed that the expression levels of HSPB8 were significantly upregulated in exosomes released by MDA-MB-231 after hyperthermia (Fig. 6A). Therefore, macrophages were transfected with HSPB8-OE plasmid and subcultured for 48 h in fresh medium (Fig. 6B). The polarization changes were evaluated by examining the levels of mRNA markers in M1 and M2 phenotype using RT-qPCR. The expression levels of IL-12 and iNOS in macrophages were significantly upregulated in macrophages transfected with HSPB8-OE, while the expression levels of the CD206 and ARG1 were significantly downregulated (Fig. 6C). Immunofluorescence results confirmed that the overexpression of HSPB8 upregulated M1 TAM protein CD86 (Fig. 6D). Additionally, flow cytometry revealed that the proportion of M1 macrophages was upregulated when macrophages were transfected with HSPB8-OE, whereas that of M2 macrophages was downregulated (Fig. 6E).

**Fig. 7:**
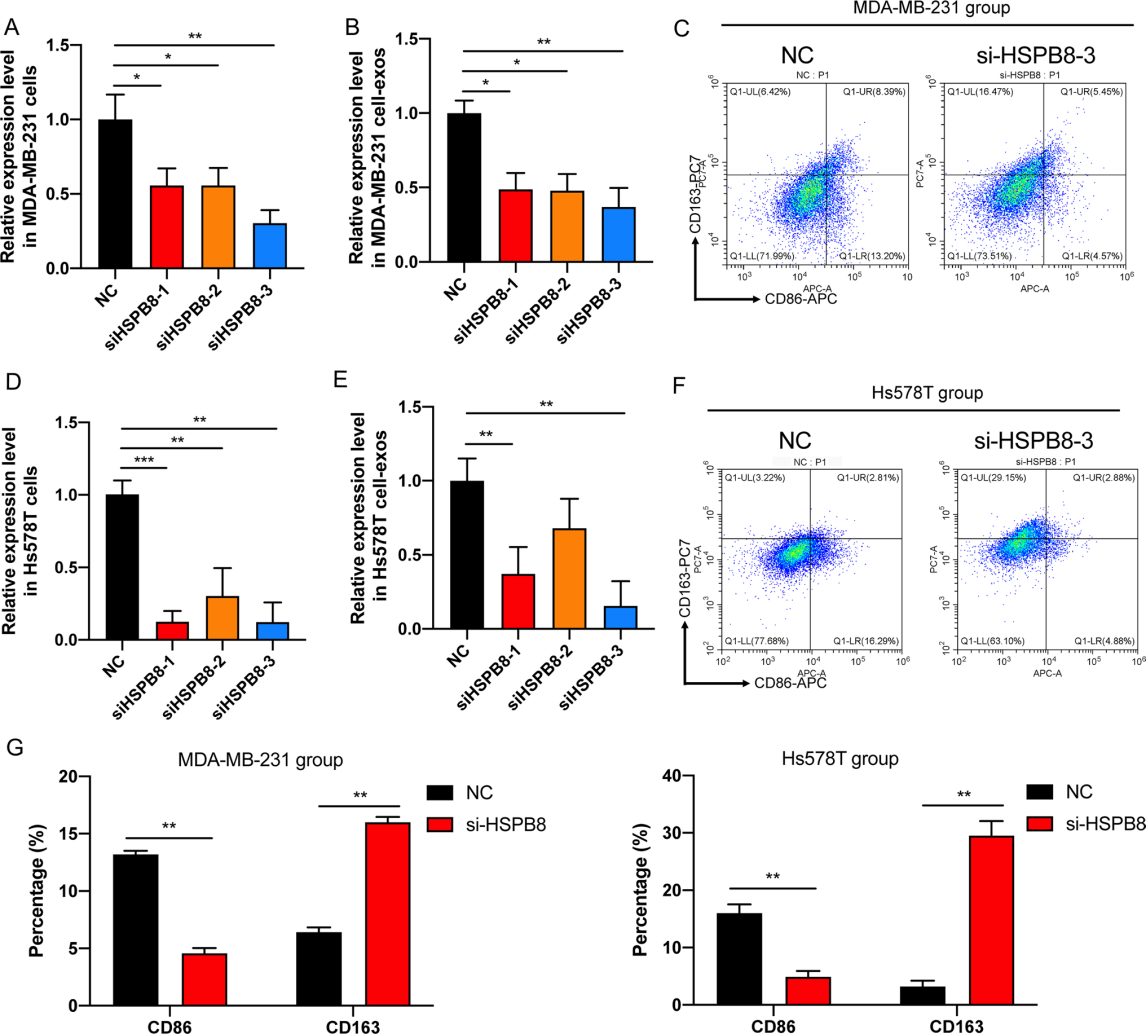
HSPB8 knockdown inhibits the M1 polarization in macrophages. **A** The transfection efficiency of si-HSPB8 in pre-hyperthermia treated MDA-MB-231 cells were tested by RT-qPCR. **B** The expression level of HSPB8 in exosomes derived from pre-hyperthermia treated MDA-MB-231 cells transfected with si-HSPB8 or NC. **C** The proportions of CD86 + M1 TAM and CD163 + M2 TAM were analyzed by flow cytometry. **D** The transfection efficiency of si-HSPB8 in pre-hyperthermia treated Hs578T cells were tested by RT-qPCR. **E** The expression level of HSPB8 in exosomes derived from pre-hyperthermia treated Hs578T cells transfected with si-HSPB8 or NC. **F** The proportions of CD86 + M1 TAM and CD163 + M2 TAM were analyzed by flow cytometry. **G** The statistical data of proportions were analyzed by GraphPad Prism 8.0

Furthermore, to verify the effect of HSPB8 on hyperthermia-mediated M1 polarization, we conducted HSPB8 knockdown experiments in pre-hyperthermia treated MDA-MB-231 and Hs578T cells and then co-cultured macrophages with exosomes derived from transfected MDA-MB-231 and Hs578T cells. RT-qPCR analysis showed that the expression levels of HSPB8 were downregulated both in cells and exosomes released by pre-hyperthermia treated MDA-MB-231 and Hs578T cells, with the si-HSPB8-3 downregulated the most significantly (Fig. 7A–D). Flow cytometry revealed that the proportion of M1 macrophages was downregulated when macrophages were co-cultured with exosomes derived from pre-hyperthermia treated MDA-MB-231 and Hs578T cells transfected with si-HSPB8-3 compared with NC, whereas that of M2 macrophages was upregulated. Therefore, we concluded that HSPB8 is an important downstream factor in hyperthermia-mediated action to promote M1 polarization.

## Discussion

Owing to the lack of targeted therapies, the clinical outcomes of TNBC are poorest in all types of breast cancer [26]. Therefore, verifying novel therapeutic targets may improve the prognosis of patients with TNBC. This study demonstrated that hyperthermia is an alternative treatment method for TNBC and that it exerts therapeutic effects by regulating cell cycle and apoptosis. Hyperthermia has been believed to be able to transform poorly immunogenic or non-immunogenic tumor cells into high immunogenic ones. Besides directly killing cancer cells, hyperthermia was reported to remodel the immune compartment of TME [27]. However, molecular mechanisms underlying the effect of hyperthermia in TME still remains elusive.

Exosomes contributed significantly to the intercellular communication between breast cancer and metastasis microenvironment [28]. Exosomes can promote the distant tumor nourishing environment and form the pre-metastasis niche [29, 30]. This study demonstrated that hyperthermia can rapidly promote the abundant secretion of exosomes, which lays foundation for our follow-up exploration on the function and mechanism of exosomes in the TME. The low immunogenicity of exosomes enabled them to freely pass through the tissue barrier in the TME, carry target genes into the receptor cells, and release genes to exert their functions [31]. Compared with other cells, macrophages have the strongest phagocytic ability. Thus, we speculated that exosomes released by breast cancer cells after hyperthermia are taken up mainly by macrophages. Therefore, we hypothesized that hyperthermia could affect tumor immune microenvironment by promoting the release of large amounts of exosomes and change the cargo contents in the exosomes.

The genome-wide gene data demonstrated an enrichment of MAPK, TNF, and IL-17 signaling pathways. Among them, MAPK signaling pathway is reported to be associated with exosome secretion [32]. The MAPK, TNF, and IL17 signaling pathways are classical inflammation-related pathways. Wang et al. found that photodynamic therapy (PDT) could promote the polarization of M1 macrophage through the ERK/MAPK pathway [33].Also, the upregulation of MAPK phosphorylation level increased IL1, IL6, and TNF. The TNF pathway was a classic M1 type macrophage-related pathway. Studies found that macrophages can drive TNF-induced cell death through TRIF/CD14 [34]. Therefore, we hypothesized that hyperthermia can inhibit tumor progression and promote macrophage reprogramming through inflammation-related pathways. On these bases, our RT-qPCR results revealed that heat shock proteins (HSPs) family members, including HSPA1A, HSPA1B, HSPA6 and HSPB8 significantly increased in both cells and exosomes after hyperthermia. Some HSPs have been identified to play both positive and negative effects with immune modulation after released into the extracellular environment [35]. Fagone et al. used oligonucleotide microarray in the process of macrophage polarization and discovered 6 upregulated HSPs transcripts in M1 compared with M0 macrophages, including HSPBAP1, HSPA5, HSP90B1, DNAJB1, HSPA6 and DNAJC9 [36]. Several studies found that mild hyperthermia could release a large amount of damage-associated molecular patterns (DAMP), including heat shock protein 70 (HSP70), to effectively trigger a systemic antitumor immune response [37, 38].

Next, the function of HSPB8, which exhibited the highest upregulation, was examined. The effect of exosomal HSPB8 secreted by hyperthermia-treated TNBC cells on TAMs was examined using immunofluorescence and flow cytometry. Exosomal HSPB8 released from TNBC cells after hyperthermia were found to reprogram the phenotype of macrophages into M1 phenotype. HSPB8, also called small stress protein-like protein (sHSP22), belongs to the heat shock protein small family HSPB (HSP27) and plays important roles in cell degradation pathways, cell division machinery, and inflammation [39]. The expression of HSPB8 varies in different types of cancer cells. Additionally, HSPB8 has contrasting roles in different cancers. Rie et al. reported that HSPB8 can repress hepatocellular carcinoma progression by downregulating the PI3K/AKT signaling, while Yu et al. demonstrated that HSPB8 promoted the migration of lung adenocarcinoma cells by maintaining mitochondrial function [40, 41]. The expression of HSPB8 in healthy breast tissue was higher than that in breast cancer tissues. Additionally, patients with breast cancer exhibiting upregulated expression of HSPB8 were associated with improved prognosis In our study, we found that HSPB8 were upregulated in exosomes secreted by breast cancer cells after hyperthermia. This study also identified the novel mechanisms of exosomes involved in regulating M1 macrophage polarization via HSPB8.

Hyperthermia is an emerging tumor treatment strategy, which has been applied alone or as an adjunctive with other anti-cancer therapeutics. The common techniques of clinical hyperthermia include radio frequency, microwave, ultrasound, laser, magnetic field, hot water bath and infusion. To apply thermal therapy to breast cancer treatment, researchers are keeping on developing new techniques. Yu et al. designed an innovative graphene-based flexible device to produce far-infrared ray, which was utilized as a hyperthermia technique for TNBC treatment. Benefit from the similarity between the emission spectra of their device and the absorption spectra of the living tissue, the in vivo study demonstrated a 42% tumor growth rate inhibition, which is obviously higher than that of traditional far-infrared hyperthermia treatment [42]. In our previous study, we have demonstrated that mild hyperthermia helped sensitize the breast cancer resistant cells (MCF-7/ADR) to doxorubicin (DOX) [24]. Wang et al. compared photothermal therapy using pulse laser-triggered localized heat and water bath hyperthermia in treating MCF-7/ADR cells. Their data show that photothermal therapy exhibited much better performance by both enhancing sensitization to DOX and inhibiting DOX efflux [43].

In clinical practice, thermal ablation (≥ 60 °C) taking use of radio frequency, laser and microwave has been used in breast cancer treatment. Using scRNA-seq, Zhou et al. characterized the peripheral immune response of 6 patients with early-stage breast cancer induced by microwave ablation. The data indicate NK and T cells were activated after microwave ablation. Moreover, in vitro study showed that both CTLA-4 inhibitor KN044 and PD-1 inhibitor camrelizumab synergistically activated the peripheral T cells obtained from 7 patients after microwave ablation [44]. Mile temperature hyperthermia (≈ 42 °C) is believed to be able to improve clinical efficacy of radiotherapy and used to treat recurrent breast cancer. The biological mechanisms underlying the radiosensitizing effect of hyperthermia may be heat-mediated oxygenation and immune response [45]. These clinical data inspire us that the elevated temperature may remodel tumor immune microenvironment.

Tumor heterogeneity and the complexity of the TME are major hurdles for tumor therapy. Tumor cells need to escape the monitoring and inhibition of the immune system to survive. In the immune microenvironment, TAMs are important immune stromal cells and have critical roles in the dynamic transformation from anti-tumor functions to pro-tumor functions. M1-type TAMs, which are mainly activated by IFN-γ, LPS, and CSF1, present antigens and exert cytotoxic effects on cancer cells [46, 47]. A low M1/M2 ratio predicts poor prognosis of patients [20]. Thus, therapeutic strategies targeting macrophages are getting more attention. Based on the complex functions of TAMs in breast cancer, reprogramming M2 macrophages into M1 macrophages can inhibit tumor growth and tumor immune escape. For example, Ramesh et al. modeled a self-assembled double inhibitor DNT, which can effectively repolarize M2 macrophages into active M1 phenotype in the invasive breast cancer mouse model [48]. Zoledronic acid can promote M1 macrophage polarization for the treatment of bone metastasis of breast cancer [49].

The clinical hyperthermia apparatus is usually composed by a microwave unit and an applicator [50]. Clarifying the molecular mechanism can help determine the most appropriate technique of hyperthermia treatment planning. Here we carried out hot water bath treatment to exploit the in vitro effects of hyperthermia on immune microenvironment. However, there is still a lack of in vivo studies using hot water bath for its poor heat conductivity in living tissue. In future research, we will use microwave hyperthermia to conduct an in vivo study to further confirm the mechanism. Altogether, our data indicates subjecting “cold” tumors such as TNBC to hyperthermia can markedly improve the anti-tumor immune effect, which is the future direction of immunotherapy development.

## Conclusion

**Fig. 8:**
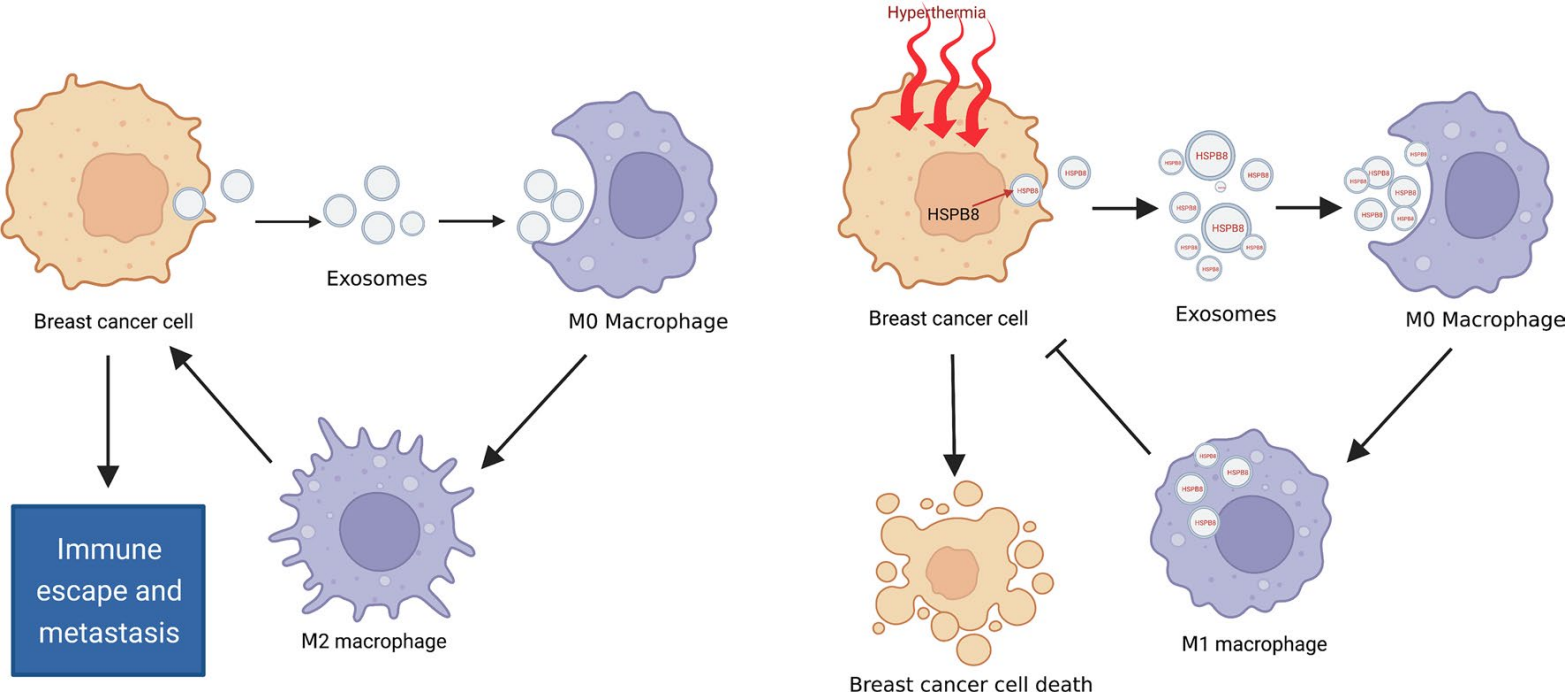
Schematic illustration of hyperthermia promotes M1 polarization of macrophages via exosome-mediated HSPB8 transfer in triple negative breast cancer. Created with BioRender.com.

This study revealed the growth-inhibitory effects of hyperthermia treatment against TNBC cells and indicated that hyperthermia can induce M1 polarization of macrophages via exosome-mediated HSPB8 transfer, which may remodel the tumor immune microenvironment of TNBC (Fig. 8). The results will help with future development of an optimized hyperthermia treatment regime for clinical application, especially for combination treatment with immunotherapy. Of course, it is still necessary to take into account the other immune cells and carry out the optimal treatment time intervals.

## Electronic supplementary material

Supplementary Material 1

## Data Availability

The data underlying this article will be shared on reasonable request to the corresponding author.
