# Supplementary material for: Hyperthermia promotes M1 polarization of macrophages via exosome-mediated HSPB8 transfer in triple negative breast cancer
Source: Discov Oncol. 2023 May 26;14:81. doi: 10.1007/s12672-023-00697-0 (PMC10219922; doi:10.1007/s12672-023-00697-0)
Supplement: Supplementary file 1 — Supplementary Material 1 [file 12672_2023_697_MOESM1_ESM.docx]

**Supplement Figure 1 Effect of hyperthermia on different non-tumor cells.**

**
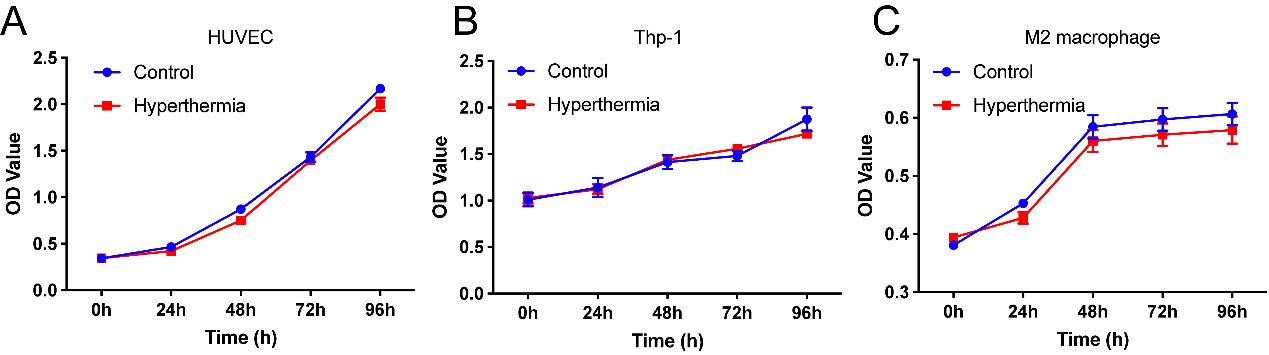
**

(A) HUVEC cells, (B) Thp-1 cells, (C) M2 macrophage cells were treated with 43°C water bath for 1 h. Cell viability was measured by using the CCK-8 assay.

**Supplement Figure 2 Expression and overall survival of FOS, FOSB, EGR1 and EGR3 in breast cancer and their correlation with macrophages.**


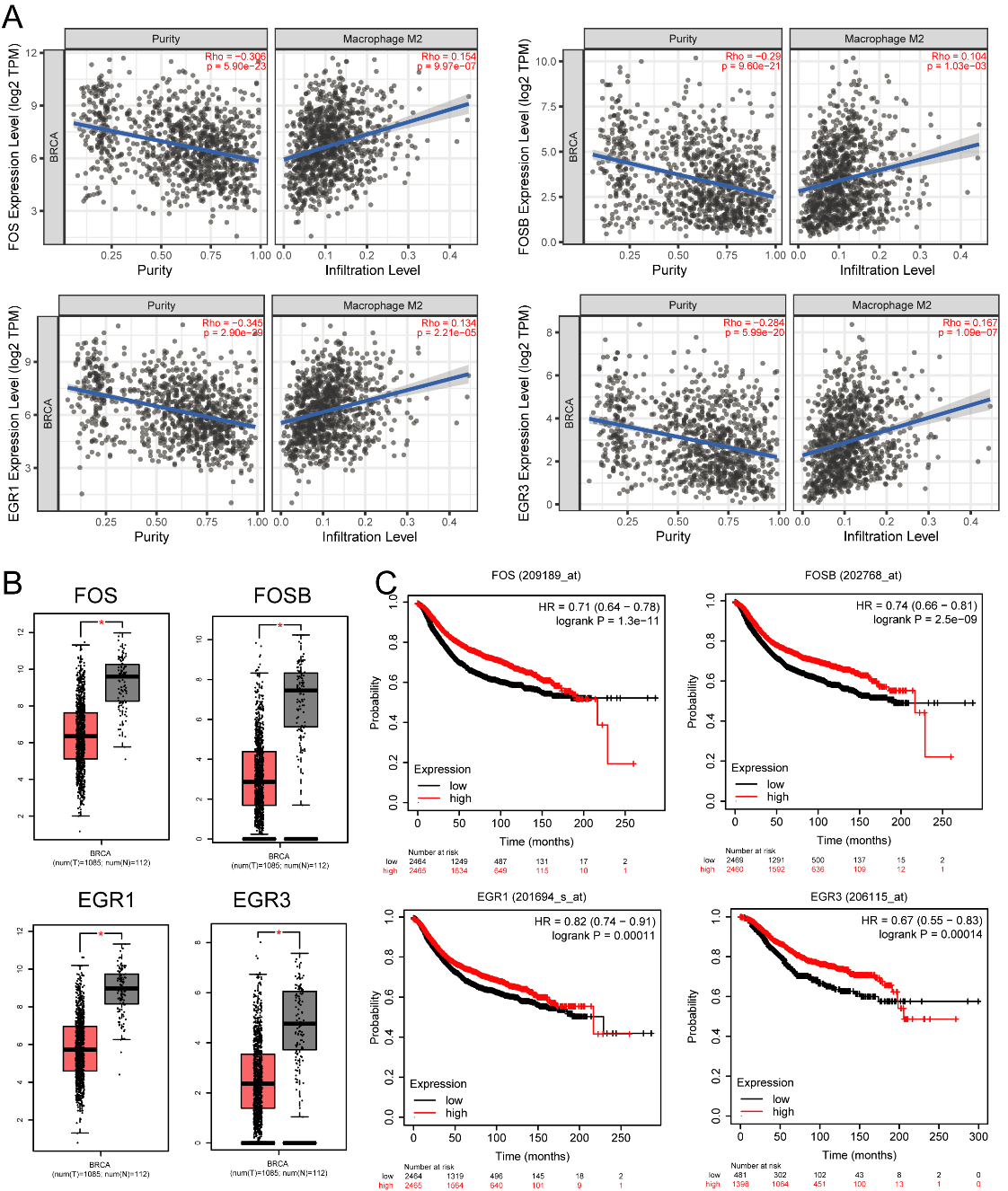


(A) The correlation of FOS, FOSB, EGR1 and EGR3 expression with macrophages infiltration by TIMER2.0 database. (B) Different expression of FOS, FOSB, EGR1 and EGR3 between invasive breast cancer and normal breast tissue by TCGA database. (C) Kaplan–Meier curves showing overall survival of FOS, FOSB, EGR1 and EGR3 in breast cancer by TCGA database.

**Supplement figure 3 Expression and overall survival of HSPB8 in breast cancer**


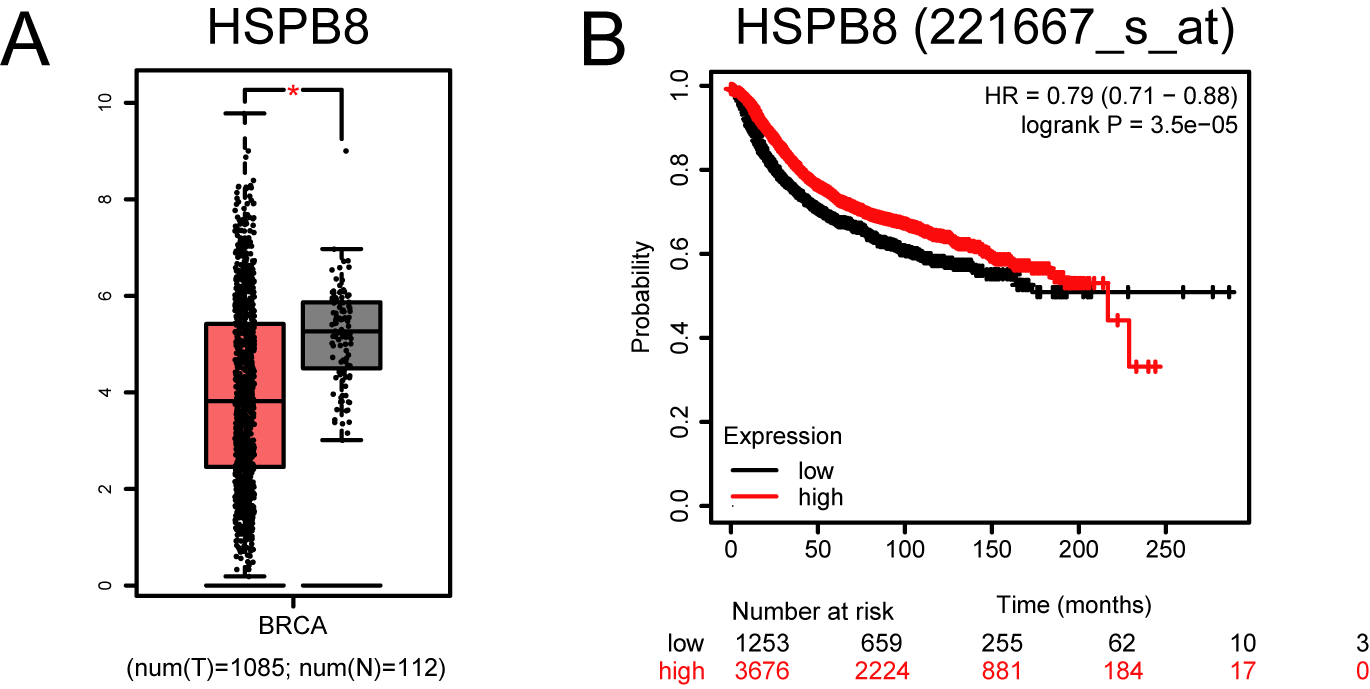


(A) Different expression of HSPB8 between invasive breast cancer and normal breast tissue by TCGA database. (B) Kaplan–Meier curves showing overall survival of HSPB8 in breast cancer by TCGA database.

**Supplementary Figure 4. Uncropped western blot bands.**

**
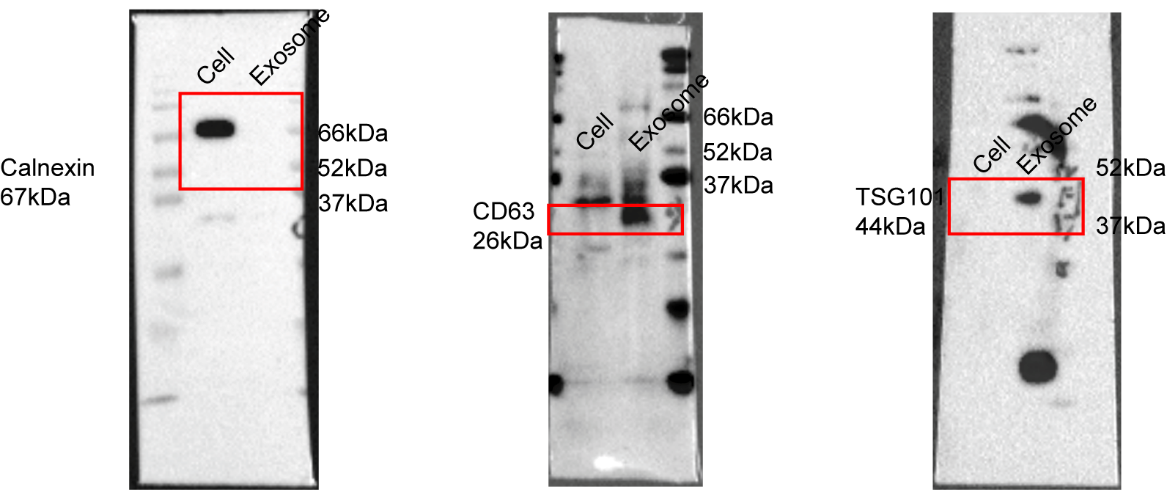
**

**Supplement Table 1 Sequences of forward primers and reverse primers using for RT-qPCR**

| **mRNA ID** | **Forward Primer (5′-3′)** | **Reverse Primer (5′-3′)** |
| --- | --- | --- |
| IL12 | TGCCCATTGAGGTCATGGTG | CTTGGGTGGGTCAGGTTTGA |
| iNOS | AGGGACAAGCCTACCCCTC | CTCATCTCCCGTCAGTTGGT |
| CD206 | TCCGGGTGCTGTTCTCCTA | CCAGTCTGTTTTTGATGGCACT |
| ARG1 | GTGGAAACTTGCATGGACAAC | AATCCTGGCACATCGGGAATC |
| TNF | GAGGCCAAGCCCTGGTATG | CGGGCCGATTGATCTCAGC |
| FOS | CTTCCCAGAAGAGATGTCTGTG | TGGGAACAGGAAGTCATCAAAG |
| CXCL10 | GTGGCATTCAAGGAGTACCTC | TGATGGCCTTCGATTCTGGATT |
| CCL5 | CCAGCAGTCGTCTTTGTCAC | CTCTGGGTTGGCACACACTT |
| EGR1 | GGTCAGTGGCCTAGTGAGC | GTGCCGCTGAGTAAATGGGA |
| IL31 | CACGTTGCCCGTCCGTTTA | TCTTCGAGAGGGACTGTAATTCC |
| FOSB | GCTGCAAGATCCCCTACGAAG | ACGAAGAAGTGTACGAAGGGTT |
| HSPA1A | GCCTTTCCAAGATTGCTGTT | TCAACATTGCAAACACAGGA |
| HSPB8 | CTCCTGCCACTACCCAAGC | GGCCAAGAGGCTGTCAAGT |
| CRYAB | AGGTGTTGGGAGATGTGATTGA | GGATGAAGTAATGGTGAGAGGGT |
| HSPA6 | CATCGCCTATGGGCTGGAC | GGAGAGAACCGACACATCGAA |
| ARC | AGCGGGACCTGTACCAGAC | GCAGGAAACGCTTGAGCTTG |
| IL17F | GGGCTGCATCAATGCTCAAG | CGGACGACCAGGGTCTCTT |
| HSPA1B | TTTGAGGGCATCGACTTCTACA | CCAGGACCAGGTCGTGAATC |
| EGR3 | GACATCGGTCTGACCAACGAG | GGCGAACTTTCCCAAGTAGGT |
| GAPDH | GGAGCGAGATCCCTCCAAAAT | GGCTGTTGTCATACTTCTCATGG |

**Supplement Table 2 List of the top 20 up-regulated genes by hyperthermia treatment in MDA-MB-231 cells**

| **Gene.Symbol** | **Fold_Change** | **P.Value** | **adj.P.Value** |
| --- | --- | --- | --- |
| HSPA6 | 12.34220903 | 4.54E-45 | 9.82E-42 |
| CRYAB | 10.5030895 | 1.79E-146 | 2.20E-142 |
| RNA5-8SN1 | 9.602866065 | 6.26E-06 | 0.0002807 |
| TDRG1 | 9.393690823 | 2.53E-26 | 2.45E-23 |
| AC025031.2 | 8.895956973 | 4.16E-12 | 7.98E-10 |
| XIRP1 | 8.849738359 | 1.16E-36 | 1.85E-33 |
| ARC | 8.836556832 | 5.51E-157 | 1.01E-152 |
| ID2 | 8.6451324 | 6.97E-125 | 6.41E-121 |
| FOSB | 8.553101371 | 5.63E-56 | 1.88E-52 |
| CACNA1G | 8.415690485 | 7.07E-24 | 5.91E-21 |
| AC022217.1 | 8.402101777 | 1.48E-21 | 9.74E-19 |
| GADD45G | 8.355470034 | 8.87E-44 | 1.72E-40 |
| TRPM5 | 8.300633724 | 7.40E-11 | 1.14E-08 |
| CHRNA2 | 8.266636689 | 1.87E-08 | 1.74E-06 |
| AC017104.2 | 8.24819595 | 2.97E-17 | 1.19E-14 |
| TRIM72 | 8.047849271 | 1.64E-12 | 3.43E-10 |
| SBSN | 8.035479735 | 9.86E-16 | 3.30E-13 |
| IGBP1P4 | 8.014757981 | 2.04E-14 | 5.56E-12 |
| HSPA1A | 8.00080639 | 1.00E-54 | 3.07E-51 |
| C6orf222 | 7.960783692 | 1.55E-13 | 3.68E-11 |

**Supplement Table 3 List of the top 20 down-regulated genes by hyperthermia treatment in MDA-MB-231 cells**

| **Gene.Symbol** | **Fold_Change** | **P.Value** | **adj.P.Value** |
| --- | --- | --- | --- |
| TMPRSS11D | -5.804838735 | 5.86E-05 | 0.001813926 |
| SFTPA2 | -5.710346935 | 0.001522173 | 0.023270212 |
| AC123768.3 | -5.493757568 | 0.000184712 | 0.004648997 |
| AL137847.2 | -5.481835882 | 0.000285549 | 0.006554799 |
| MIR7856 | -5.102469556 | 0.001059927 | 0.017441833 |
| AC079298.3 | -4.998001136 | 0.001639012 | 0.024666959 |
| LINC01252 | -3.986506741 | 0.000124554 | 0.003379958 |
| LYPLAL1-AS1 | -3.931224085 | 0.00015773 | 0.004095971 |
| SEC22B3 | -3.740001759 | 0.001229167 | 0.019707516 |
| SNORA47 | -3.580091321 | 5.35E-05 | 0.001681594 |
| KLRC3 | -3.55057732 | 0.003026321 | 0.038136824 |
| SLCO1A2 | -3.371441453 | 4.64E-07 | 3.00E-05 |
| AC104137.1 | -3.367570012 | 0.000334027 | 0.007431193 |
| RN7SL192P | -3.28802649 | 0.000999283 | 0.016698738 |
| AL035603.1 | -3.21285744 | 0.00044089 | 0.009150263 |
| AL022098.1 | -3.091795363 | 0.001792124 | 0.026231018 |
| AC023794.5 | -3.089066096 | 0.000875778 | 0.01512957 |
| RPS20P31 | -3.077056104 | 0.001561998 | 0.023765906 |
| AC096719.1 | -3.030669433 | 0.003569709 | 0.043052958 |
| AL355578.1 | -2.988722198 | 0.00312896 | 0.039069 |
